# Nano-(Q)SAR for Cytotoxicity Prediction of Engineered Nanomaterials

**DOI:** 10.3390/molecules24244537

**Published:** 2019-12-11

**Authors:** Andrey A. Buglak, Anatoly V. Zherdev, Boris B. Dzantiev

**Affiliations:** 1 A. N. Bach Institute of Biochemistry, Research Center of Biotechnology, Russian Academy of Sciences, Leninsky Prospect 33, 119071 Moscow, Russia; zherdev@inbi.ras.ru (A.V.Z.); boris.dzantiev@mail.ru (B.B.D.); 2 Physical Faculty, St. Petersburg State University, 7/9 Universitetskaya Naberezhnaya, 199034 St. Petersburg, Russia; 3 Institute of Physiologically Active Compounds, Russian Academy of Sciences, Severny Proezd 1, 142432 Chernogolovka, Moscow Region, Russia

**Keywords:** engineered nanomaterials, safety of nanomaterials, toxicological tests, modeling, descriptors, quasi-QSAR

## Abstract

Although nanotechnology is a new and rapidly growing area of science, the impact of nanomaterials on living organisms is unknown in many aspects. In this regard, it is extremely important to perform toxicological tests, but complete characterization of all varying preparations is extremely laborious. The computational technique called quantitative structure–activity relationship, or QSAR, allows reducing the cost of time- and resource-consuming nanotoxicity tests. In this review, (Q)SAR cytotoxicity studies of the past decade are systematically considered. We regard here five classes of engineered nanomaterials (ENMs): Metal oxides, metal-containing nanoparticles, multi-walled carbon nanotubes, fullerenes, and silica nanoparticles. Some studies reveal that QSAR models are better than classification SAR models, while other reports conclude that SAR is more precise than QSAR. The quasi-QSAR method appears to be the most promising tool, as it allows accurately taking experimental conditions into account. However, experimental artifacts are a major concern in this case.

## 1. Introduction

Nanomaterials and nanoparticles (NPs) possess unique physico-chemical properties (size, shape, chemical composition, physiochemical stability, crystal structure, surface area, surface energy, and surface roughness [1]), which give them beneficial characteristics. For this reason, nanotechnology is a new and rapidly growing field of knowledge which includes design, development, and usage of NPs and nanomaterials. According to the Organization for Economic Co-operation and Development (OECD), there exist 11 types of engineered nanomaterials (ENMs): Cerium oxide, dendrimers, fullerenes, gold nanoparticles, multi-walled carbon nanotubes (MWCNTs), nanoclays, silicon dioxide, silver nanoparticles, single-walled carbon nanotubes (SWCNTs), titanium dioxide, and zinc oxide.

The toxicity of ENMs and their influence on humans and the environment should be carefully evaluated [2,3]. Generally, there are five key mechanisms of ENMs’ toxicity: (1) Direct lesion by ion detachment; (2) oxidative stress induced by reactive oxygen species; (3) adsorption of biologically active molecules; (4) photochemical and redox reactions; and (5) Trojan horse effects (NPs may act as vectors for the transport of toxic compounds into cells) [4,5,6,7,8]. Not only is complete experimental characterization of the toxicity for all varying preparations extremely laborious, but predictions of the theoretical descriptions of the correspondence between structure/composition of ENMs and their biological activity are in demand.

**Figure 1:**
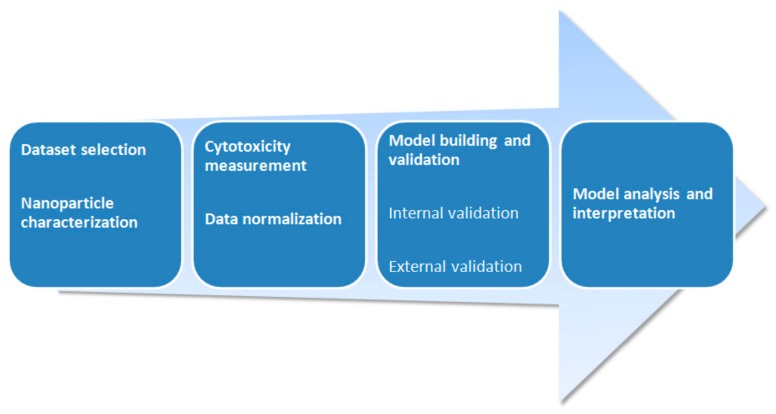
A typical workflow of QSAR modeling for nanoparticles (NPs).

Quantitative structure-activity relationship, or QSAR (Figure 1), is an area of molecular modeling that studies relationships between structure and activity using mathematical statistics and machine learning methods. QSAR is efficiently used to predict toxicity of chemical substances [9,10,11,12,13]. Classical QSAR is a so-called Hansch analysis [14], which stands on the assumption that bioactivity of compounds is correlated with geometrical and physicochemical descriptors. Generally, a molecular descriptor can be considered as a “number” describing a certain molecular property, which might be experimentally determined (i.e., dipole moment) or calculated (i.e., potential energy), or determined from the chemical structure (i.e., number of methyl groups). However, a molecular descriptor may be a mathematically obtained property (i.e., Wiener, Balaban, or Randic indices)—chemical graph theory is often used to derive mathematical descriptors [15]. Three-dimensional QSAR is another approach which allows building relations between the spatial structure of molecules, interaction fields, and activity. The first application of the three-dimensional (3D) QSAR technique was proposed in 1988 by Cramer and co-authors [16], when they were first to develop comparative molecular field analysis (CoMFA). CoMFA supposes that differences in bio-activity depend on the change of strength of non-covalent interaction fields (electrostatic and van der Waals) around the molecules. Another 3D QSAR method is comparative molecular similarity indices analysis (CoMSIA), which takes into account the same molecular interactions as CoMFA, but with the addition of hydrophobic interactions and hydrogen bonding. CoMSIA was developed in 1994 [17]. Three-dimensional QSAR provides multiple benefits to a researcher who studies organic compounds. However, 3D QSAR and classical molecular descriptors are unable to express the specificity of nanoparticles, because their exact structure is usually unknown. This circumstance leads to a lack of sufficient molecular descriptors appropriate for nano-QSAR modeling [18].

**Figure 2:**
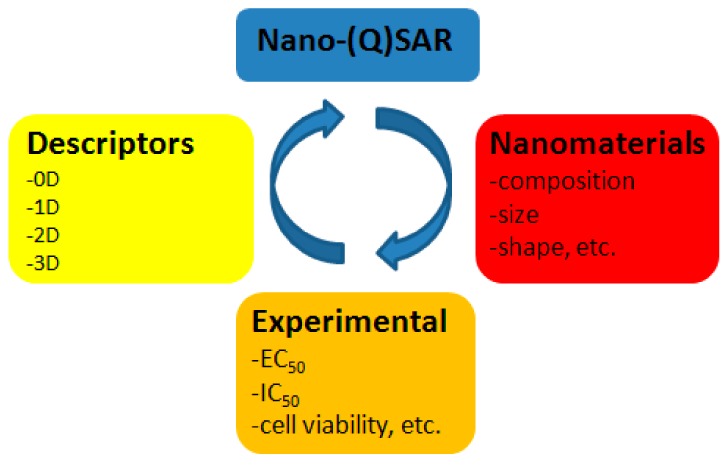
A general scheme of nano-(Q)SAR modeling. 0D, zero-dimensional; 1D, one-dimensional; 2D, two-dimensional; 3D, three-dimensional.

Nano-QSAR (Figure 2) allows the efficient study of nanoparticles and determination of correlations between their structure and activity [19]. Nano-QSAR may use all three approaches: One-dimensional (1D), two-dimensional (2D), and 3D QSAR [20,21,22]. However, it also raises a question: Which technique (nano-Hansch, nano-CoMFA, or nano-CoMSIA) is the best way to study nano-objects? There have been attempts to answer this question. Jagiello and co-authors compared the performance of nano-QSAR and 3D nano-QSAR, studying the activity of fullerene derivatives [23]. They concluded that nano-QSAR is a more universal approach, which allows gathering general information about the mode of biological activity of nanomaterials: Not only the receptor-based response, but also cell- and organism-based responses. The latter allows efficiently predicting the toxicity of nanoparticles. However, the application of 3D QSAR should be used to study the receptor-based response and would help in understanding such activity in detail [23]. In general, application of QSAR modeling of nanomaterials can reduce the need for time- and labor-consuming cytotoxicity tests, which are extremely important and economically feasible.

There are several classes of theoretical molecular descriptors: 0D-descriptors (for example, constitutional and count descriptors), 1D descriptors (for example, structural fragments, fingerprints), 2D descriptors (graph invariants), and 3D descriptors (quantum-chemical descriptors, size, steric, surface, and volume descriptors). Molecular descriptors cannot be determined for super-complex substances such as NPs and ENMs, since clear representation of their molecular structure is usually absent. In this regard, the basic idea is to change the traditionally used paradigm of “the endpoint is a mathematical function of the molecular structure”, to another paradigm: “The endpoint is a mathematical function of available eclectic information”. The eclectic data may include experimental data and can be (1) conditions of a synthesis, (2) technological attributes, (3) size of nanoparticles, (4) concentration, and (5) attributes related to cell membranes, etc. Such an approach is called quasi-QSAR [24].

In this respect, the aim of the present review is to summarize all available data on nano-QSAR usage for cytotoxicity predictions of nanomaterials and nanoparticles. In addition, we attempt to analyze the efficiency of 2D and 3D QSAR in studying nanomaterials, to compare which technique is the best for each class of nanoparticles: Fullerenes, metal oxides, metal nanoparticles, etc. We also critique recent papers on the usage of nano-QSAR for cytotoxicity research. This review summarizes exactly ten years of experience since the appearance of the first paper [25] studying the cytotoxicity of nanomaterials with the (Q)SAR technique. We establish here classes of NPs with available data for (Q)SAR consideration: Metal oxides, metal-containing particles, MWCNTs, fullerenes, and silica.

## 2. Metal Oxides

Metal oxide NPs are used in renewable energy, wastewater treatment, electronics, cosmetics, textiles, foods, agriculture, medicine, pharmaceutics, and for many other purposes. Metal oxides are probably the most well-studied object of nano-QSAR research. The pioneer work by Hu et al. investigated seven nano-sized metal oxides: ZnO, CuO, Al_2_O_3_, La_2_O_3_, Fe_2_O_3_, SnO_2_, and TiO_2_. They applied the multiple linear regression (MLR) method. The cytotoxicity towards *Escherichia coli* was found to be highly correlated with metal cation charge. The higher the cation charge, the lower the cytotoxicity of the nano-sized metal oxide [25]. The cytotoxicity of metal oxide ENMs were measured in terms of LD_50_: The dosage of NPs shown to cause the death of 50% of *E. coli* cells. 

The oxidative stress potential of metal oxide NPs could be predicted by looking at their band gap energy [5]. Puzyn and co-authors developed a model describing the cytotoxicity towards *Escherichia coli* of nanoparticles based on 16 different metal oxides and SiO_2_ [20]. All quantum-chemical calculations were performed using the PM6 semi-empirical method. They applied the MLR method combined with a genetic algorithm. The model obtained was characterized by R^2^ = 0.862. The model reliably predicted the toxicity of all metal oxides and included only one descriptor—ΔH_Me+_—which is the enthalpy of formation of a gaseous cation. The endpoint of cytotoxicity measurement was LD_50_. Log(1/LD_50_) was used as a dependent variable in the MLR equation.

The structure–cytotoxicity relationship for the same dataset of 17 metal oxide NPs was further investigated in a succession of papers [18,26,27,28,29,30,31,32]. Density functional theory (DFT)-based descriptors (energy gap, hardness, softness, electronegativity, and electrophilicity index), in conjunction with the MLR statistical method, were used to find a high correlation between experimental and predicted activity values [27]. The absolute electronegativity is defined as half of the summation between the ionization potential and the electron affinity. The absolute hardness is defined as half the difference between the ionization potential and the electron affinity. Within the Koopmans’ theorem approximation, these parameters can be expressed as the highest occupied molecular orbital (HOMO) and lowest unoccupied molecular orbital (LUMO) energies. Thus, electronegativity (χ) is determined according to the equation:

(1) $$\chi \;=\;-\;\frac{HOMO+LUMO}{2}$$

Hardness (η) is determined according to the equation:

(2) $$\eta =-\;\frac{HOMO-LUMO}{2}$$

In a model by Kar et al., electronegativity (χ) and charge of the metal cation were used as molecular descriptors to build QSAR models for the prediction of cytotoxicity of metal oxide NPs (Table 1). They hypothesized that small particles of metal oxides release an electron much easier than the same particles in the crystal structure; small fragments initiate formation of reactive oxygen species, which invoke the oxidative stress condition to bacteria [28]. A simple QSAR model with high predictive ability (R^2^ = 0.87) was built based on two descriptors: Absolute electronegativity of metal and electronegativity of metal oxide [32]. In addition, a high correlation (R^2^ = 0.804) was obtained to predict the photo-toxicity of metal oxide NPs using two descriptors: Molar heat capacity and LUMO energy of the metal oxide [32]. The best model by Mu et al. associated cytotoxicity of 16 metal oxide NPs towards *E. coli* with enthalpy of formation of a gaseous cation (ΔH_me+_) and polarization force (Z/r) [33]:

Log(1/EC_50_) = (4.412 ± 0.165) + (−0.121 ± 0.068) Z/r + (0.001 ± 2.57 × 10^−4^)ΔH_me+_ (3)

The model by Pan et al. used the same dataset, a simplified molecular input line entry system (SMILES)-based optimal descriptor and the MLR method, and showed the highest predictive ability towards both training (R^2^ = 0.89–0.98) and test set (R^2^ (test) = 0.82–0.87) [18]. Other works [20,27,28,32,33] also used the MLR method.

Classification models were developed using Monte Carlo modeling [26], random forest (RF) [29,39], the ensemble learning approach [30], the read-across method [31], support vector machines (SVMs) [40], and counter propagation artificial neural networks [38]. Toropov et al. used Monte Carlo optimization of correlation weights and SMILES-based optimal descriptor [26]. The detailed validation of the model with an external dataset and six splits was performed. The distribution of the R^2^ (test) values predicted was within the 0.83–0.96 range. The model was based on the information about the presence of oxygen and double bonds [26]. Liquid drop model (LDM)-based descriptors [56], van der Waals interactions, electronegativity, and metal–ligand binding characteristics contributed to the model by Sizochenko and co-authors [29]. However, this model suffered from over-fitting: It was obtained using 13 training set NPs with seven descriptors. The RF model in [39] associated cytotoxicity with oxygen in weight percentage and enthalpy of formation of a gaseous cation. Singh and Gupta built an ensemble learning model with high stability and high predictive ability using three descriptors: Oxygen percent, molar refractivity, and polar surface area [30]. Gajewicz et al. used ionization enthalpy of the detached metal atoms to classify metal oxide NPs into toxic and non-toxic NPs [31]. The counter propagation artificial neural network models were tested for the prediction of metal oxide cytotoxicity towards *E. coli* by Fjodorova and co-authors [38]. The cytotoxicity of metal oxide NPs was found to be correlated with metal electronegativity by Pauling scale, number of metal atoms in oxide, number of oxygen atoms in oxide, and charge of metal cation [38]. 

Nano-QSAR was proposed to evaluate the cytotoxicity of metal oxide NPs towards *E. coli* [40]. Six molecular descriptors were selected and calculated using the DFT-B3LYP method. Linear and nonlinear models were built using the MLR and SVM methods, respectively. The results demonstrated that both models possessed high stability and good predictive performance, yet the statistical parameters of the SVM model were slightly higher. Five quantum-chemical parameters, namely, the highest occupied molecular orbital (HOMO) energy, α-LUMO (lowest unoccupied molecular orbital) and β-LUMO energy, the average of α-LUMO and β-LUMO, and the energy gap between the frontier molecular orbitals ∆E, as well as molar heat capacity (Cp), were involved in the model. It was revealed that LUMO energy and Cp were the two key descriptors affecting the cytotoxicity of metal oxide NPs [40]. Kaweeteerawat et al. worked with a different dataset of 24 metal oxide NPs and used an SVM approach to build a classification model, with conduction band energy and hydration enthalpy (ΔH_hyd_) descriptors used to predict cytotoxicity towards *E. coli* [41]. The endpoint of cytotoxicity measurement was IC_50_, which is a half-maximal growth inhibitory concentration. 

Gajewicz and co-authors performed a joint experimental–theoretical study to develop a nano-QSAR model describing the toxicity of 18 metal oxide NPs towards human keratinocyte cell line (*HaCaT* cells); they built a genetic algorithm MLR model using enthalpy of formation of metal oxide and Mulliken’s electronegativity descriptors [42]. Sizochenko with co-authors used the same dataset, in addition to LDM-based descriptors, van der Waals interactions, electronegativity, and metal–ligand binding descriptors, to build a RF model [29]. While using the same dataset, a SMILES-based optimal descriptor allowed the building of an MLR model [18]. The RF model by Basant and Gupta associated cytotoxicity with 10-based logarithm of solubility measured in mol/L (LogS), topological polar surface area (TPSA), and Mulliken’s electronegativity.

The comparison of the toxicity of metal oxide NPs towards bacteria *E. coli* (prokaryotic organism) and *HaCaT* cells (eukaryote) revealed that, in both cases, the exposure to metal oxide NPs caused an increase in the production of reactive oxygen species, which led to oxidative stress and, subsequent, cytotoxicity. However, the authors concluded that different modes of toxic action occur between prokaryotic and eukaryotic organisms: Dissimilarities in cell morphology, surface redox activity, and the ability of metal cations to release from the NP surface [42]. Sizochenko et al. also explored experimental toxicity data of metal oxide nanoparticles to both *E. coli* and *HaCaT* cells. They developed nano-QSAR models which showed the dissimilarities in the mechanisms of toxicity of metal oxide NPs towards eukaryotic and prokaryotic cells [29]. The individual size and aggregation size were found to be the most important factors for the toxicity towards *E. coli* and *HaCaT* cells, especially for the latter [18]. It was found that cytotoxicity of metal oxide NPs towards *E. coli* and *HaCaT* are correlated. pEC_50_ (half maximal effective concentration in logarithmic form) of *E. coli* depended on the enthalpy of formation of a gaseous cation (ΔH_Me+_), charge of the metal cation (χ_ox_), and pEC_50_ of *HaCaT*, while pEC_50_ of *HaCaT* depended on the enthalpy of formation of metal oxide (ΔH_f_) nano-cluster, electronic chemical potential of the cluster, and pEC_50_ of *E. coli* [36]. Kuz’min et al. also investigated cytotoxicity of metal oxide NPs towards both *HaCaT* cells and *E. coli*. Poor applicability of classic 2D descriptors for representation of metal oxide nanoparticles was demonstrated. The combination of 1D descriptors and size-dependent descriptors was used to reveal the composition of nanoparticles. For this purpose, descriptors based on the fundamental characteristics of atoms (nuclear charge, ionization potential, electronegativity, ionic radius, etc.) were combined with descriptors obtained from the structural formula (atomic mass of the metal, charge of the nucleus of the atom, van der Waals radius of a metal, etc.), and LDM-derived size-dependent parameters (Wigner-Seitz radius R_wz_, mass density *ρ*, thickness of interfacial layer *h*, etc.) [8]. These results indicate that the specific role in cytotoxicity of metal oxide NPs is driven both by size-dependent parameters and by the chemical nature of metal ions. As was revealed in the previous studies, the main factor determining the cytotoxicity of nano-sized metal oxides is the charge of the metal ion.

A single QSAR model for predicting cytotoxicity of 16 metal oxide NPs both towards *E. coli* and *HaCaT* cells was built in [37]. The model was based on the representation of the available data, encoded into quasi-SMILES. Quasi-SMILES are an analog and an attractive alternative to more traditional SMILES. Quasi-SMILES are a tool to represent different conditions: Physico-chemical properties and experimental conditions. The statistical quality of the models was evaluated using average determination coefficient R^2^ and root mean squared error (RMSE) for the training set, which were equal to 0.79 and 0.216; R^2^ and RMSE for the validation set were equal to 0.90 and 0.247, respectively [37].

Classification cytotoxicity nano-SAR models (logistic regression) were built using a set of nine metal oxide NPs, to which transformed bronchial epithelial cells (BEAS-2B) were exposed. The best model had a 100% classification accuracy to both internal and external validation. This model was based on three descriptors: Atomization energy of the metal oxide, period of the nanoparticle metal, and nanoparticle primary size [46]. In another study, SAR models were developed using 24 metal oxide NPs towards BEAS-2B and murine myeloid (RAW 264.7) cells. Zhang et al. [6] reported regression tree models using the metal dissolution of metal oxide NPs and energy of conduction band to predict the toxicity potential of 24 metal oxide NPs. The conduction band energy was derived from the following equation with pH = 7.4 in a biological system:

E_c_ = − χ_oxide_ + 0.5E_g_ + 0.059(PZZP − pH) (4)

where E_c_ refers to conduction band energy; χ_oxide_ is an absolute electronegativity of metal oxide; E_g_ is a band gap; and PZZP is a point of zero zeta potential. 

Application of the SMILES-based optimal descriptors and quasi-SMILES (dose, exposure time) were used to build a predictive model for cell membrane damage caused by 24 metal oxide NPs towards BEAS-2B cells [49]. The experimental data were taken from the paper by Patel and co-authors [50]. The values of activity calculated with the Monte Carlo method were in good agreement with the experimental data [49]. 

A SVM approach, in conjunction with conduction band energy and ionic index (a parameter used to calculate the metal ion hydration energy, which is an indicator of the ability to form hydrated metal ions) descriptors, was used by Liu et al. The model had a high classification accuracy of 93.74% [48]. With the data from Zhang et al., Sizochenko et al. [47] built nano-SAR models for BEAS-2B and RAW 264.7 cell lines with high predictivity; they used seven and nine descriptors, respectively. The model for BEAS-2B cells included the following descriptors: Mass density, covalent index (represents interactions of NPs with protein-bound sulfhydryl and depleting glutathione), cation polarizing power (represents electrostatic interactions between NPs and cells), Wigner-Seitz radius [56], surface area-to-volume ratio and aggregation parameter (both of which are LDM-based descriptors), and tri-atomic descriptor of atomic charges (SiRMS descriptor [34,35]). The model for RAW 264.7 cell line included the following descriptors: Mass density, molecular weight, electronegativity, covalent index, surface area, surface area-to-volume ratio, two-atomic descriptor of van der Waals interactions, tetra-atomic descriptor of atomic charges, and size. As a whole, ionic, fragmental, and LDM-based descriptors revealed the structure and characteristics of metal oxide NPs [47]. A partial least squares (PLS) regression analysis was performed by Forest et al., in which 25 nanoparticles from six metal oxides with different particle sizes and shapes were synthesized and characterized. Their toxicity was evaluated using RAW 264.7 cells. A model with four chemical composition-related descriptors (metal cation charge, hydration rate, radius of the metallic cation, and Pauling electronegativity) was also built [53].

Cytotoxicity of 42 metal oxide NPs was investigated in [54]. A set of 24 TiO_2_ NPs and 18 ZnO NPs were tested for their ability to disrupt the lipid membrane in cells. Data were measured in rat L2 lung epithelial cells and rat lung alveolar macrophages. Size, concentration, size in phosphate-buffered saline, size in water, and zeta potential descriptors were used in multivariate linear regression and linear discriminant analysis (LDA)-based classification [54]. The same dataset was used by Papa et al. A total of 31 NPs was used to develop an MLR model based on three descriptors: Engineered size, size in phosphate-buffered saline (PBS), and concentration. The best model developed only for ZnO NPs was based on the same descriptors: Engineered size, size in PBS, and concentration. The best combination of variables selected to model TiO_2_ NPs was engineered size and concentration. Additionally, a simple classification model was developed, which predicted the potential for cell membrane disruption of the studied nanoparticles with good accuracy on the basis of two empirical descriptors: Experimentally determined size and concentrations. The obtained models may be beneficial to screen the potential harmful effects of nanoparticles to human and living organisms and to perform optimal design of toxicological tests [55].

Classification models for six different metal oxides and SiO_2_ were presented in [57]. The authors compared the performance of four different algorithms: Generalized linear model, SVM, RF, and neural network. The neural network model was identified as the model with the best predicting ability. The analysis of relative descriptor importance for the built neural network model identified dose, formation enthalpy, exposure time, and hydrodynamic size as the four most important descriptors [57]. However, the advantage of regression models for the analysis of toxicity of NPs was shown in comparison with the classification models on metal NPs and metal oxide NPs [58]: Regression models allow not only qualitative, but also a quantitative evaluation of the studied nanomaterials.

A quasi-QSAR model (based on quasi-SMILES descriptors) was developed to predict the cell viability of human BEAS-2B and *HaCaT* cells exposed to 21 metal oxide NPs. The cell viability data originated from six research articles [6,42,44,45,51,52]. Quasi-SMILES descriptors (core size, hydrodynamic size, surface charge, and dose) represented the physicochemical properties and experimental conditions. Hierarchical cluster analysis (HCA) and the min–max normalization method were used, and their performance compared. The quasi-QSAR model built using quasi-SMILES generated by means of HCA showed better performance than the min–max normalization method. Model quality was evaluated using adjusted determination coefficient and was shown to be satisfactory [43].

In conclusion, we have summarized the data relating to 39 QSAR and SAR models (18 for *E. coli*, nine for *HaCaT* cells, six for transformed bronchial epithelial cells (BEAS-2B), four for murine myeloid cells (RAW 264.7), and two for rat L2 lung epithelial cells and rat lung alveolar macrophages). Of the 39 models, 12 were built using the MLR method, which is reasonable since MLR has certain advantages compared to classification models [58]. Most of the descriptors in the described models relate to physico-chemical, constitutional, topological, and quantum mechanical types. The most popular descriptors are metal cation charge, electronegativity, and enthalpy of formation.

Most of the modeling for metal oxide NPs was done based on the same datasets by Puzyn and co-workers [20], Gajewicz and co-authors [42]. One can see how limited data availability is on ENMs cytotoxicity.

## 3. Other Metal-Containing Nanoparticles

In a pioneer work [59], an SVM classification model was developed using the experimental data of 44 different NPs from Shaw et al. [60]. The model used four experimentally determined descriptors: Size, zeta potential evaluating the intensity of charge on their surface, and R1 and R2 relaxivities estimating their magnetic properties. The authors concluded that QSAR is an appropriate methodology for predicting the cytotoxicity of novel nanomaterials, as well as for the design and manufacture of safer NPs. Fourches and co-authors also analyzed a dataset by Weissledder et al. [61], where cellular uptake was evaluated. They used both SVM classification and kNN regression to build predictive models. The most important descriptors were lipoplicity and a number of double bonds [59]. Yet another nano-QSAR study for the prediction of the cytotoxicity of metal-containing NPs was conducted in [62] using smooth muscle cells from Shaw et al. [60]. The model was built based on cytotoxicity data for 31 NPs using MLR and a Bayesian regularized artificial neural network. The model predicting smooth muscle apoptosis (SMA) consisted of three descriptors: Core material (I_Fe2O3_), surface coating (I_dextran_), and surface charge (I_surf.chg_):

SMA = 2.26(±0.72) − 10.73(±1.05)I_Fe_2_O_3__ – 5.57(±0.98)I_dextran_ – 3.53(±0.54)I_surf.chg_ (5)

I_Fe2O3_ was set to 1 for the Fe_2_O_3_ core and 0 when the core was Fe_3_O_4_. I_dextran_ was equal to 1 in the case of dextran coating and 0 for the others. Surface functionality was equal to 1 (basic), −1 (acidic), or 0 (neutral). The model possessed a determination coefficient for the training set equal to 0.81 and 0.86 for the test set. Table 2 summarizes the information about nano-(Q)SAR models predicting cytotoxicity of metal-containing nanoparticles.

A nano-SAR model was built allowing the classification of 44 iron core-based NPs into bioactive or inactive, using a naive Bayesian classifier based on four descriptors: Primary size, spin-lattice and spin-spin relaxivities, and zeta potential [63]. Liu et al. measured toxicity of 82 NPs against zebrafish embryo; NPs included metal and metal oxide, dendrimer, and polymeric NPs. The principal descriptors were concentration, shell composition, surface functional groups, purity, core structure, and surface charge [64].

A QSAR-perturbation model was built and predicted the cytotoxicity of NPs with an accuracy higher than 93%. Cytotoxicity against several mammalian cell lines was taken into account. The influences of molar volume, polarizability, and size of the particles were involved as principal descriptors of the model. The cytotoxicity of different silica (SiO_2_), nickel (Ni), and nickel oxide (NiO) NPs was predicted and found to be in consensus with the experiment. The dataset consisted of 1681 cases (nanoparticle-nanoparticle pairs) [65]. A perturbation model was developed for the prediction of eco- and cytotoxicity of NPs; molar volume, polarizability, size of NPs, electronegativity, hydrophobicity, and polar surface area of surface coating descriptors were included in the model. The endpoint of cytotoxicity measurement were taken from several sources: CC_50_ (cytotoxic concentration of the nanoparticle leading to 50% reduction in cell viability), EC_50_ (effective concentration of the nanoparticle that inhibits at 50% the growth of the living system), IC_50_ (concentration of the nanoparticle that inhibits the root elongation of the living system at 50%), TC_50_ (concentration that causes toxic effects in 50% of the living system), LC_50_ (lethal concentration that causes mortality in 50% of the living system). These cytotoxicity endpoint measurements were used with different cutoff values to divide NPs into two classes: Either toxic or nontoxic [66]. In their work, Kleandrova et al. developed nano-QSAR models with the aim to test the ecotoxicity of NPs on several assay organisms (bio-indicators). Ecotoxicity of three nickel-based nanoparticles was predicted. The predictions were found to be in very good agreement with the experimental evidence, confirming that Ni-nanoparticles are not ecotoxic when compared with other NPs [67]. Further, a unified in silico machine learning model based on artificial neural networks was developed by Concu and co-authors [68]; the model was aimed to simultaneously predict general toxicity profiles of NPs under diverse experimental conditions. Application of perturbation theory to a set of 260 unique NPs showed higher accuracy of more than 97%. Two families of descriptors were used in this study: Physico-chemical and 2D topological [68]. 

Global classification models were built to predict the ecotoxicity of metal core NPs. The toxicity data were retrieved from the dataset of [69]. Four tree methods (functional tree, C4.5 decision tree, random tree, and simple classification and regression trees (CART)) were used for model development. EC_50_, LC_50_, and MIC (minimum inhibitory concentration) were used as endpoints of cytotoxicity measurement. Global nano-SARs across species were shown to correctly predict more than 70% of the samples in training (320 NPs) and test sets (80 NPs). Species-specific classification models were also developed for *Danio rerio*, *Daphnia magna*, *Pseudokirchneriella subcapitata*, and *Staphylococcus aureus*. The descriptors used were molecular polarizability, accessible surface area, and solubility [70]. Species-specific models also showed high predictive ability. Boukhvalov and Yoon investigated metal NPs and developed descriptors based on the results of first-principle calculations. To estimate the activity of metal NPs, they regarded two reactions: Ion extraction from the surface of an NP to aqueous media and water dissociation on the surface. They performed calculations for a set of metals: Al, Fe, Cu, Ag, Au, and Pt. Different models of NPs were used: (001) and (111) surfaces, nanorods, and two cubic nanoparticles of 0.6 and 0.3 nm size. Significant energy dependence of the reactions from the selected model of NP was demonstrated. Descriptors revealed the dependence of chemical activity from the size and shape of nanoparticles [72].

The cytotoxicity of Au and Pd surface-modified TiO_2_-based NPs was studied using QSAR. Toxicity tests were done on *E. coli* and Chinese hamster ovary (CHO-K1) cells. Three types of clusters were investigated: Monometallic (Au, Pd) clusters, core-shell particles, and alloy bimetallic clusters (Au/Pd). The cytotoxic effect of monometallic Au-TiO_2_, Pd-TiO_2_, and bimetallic Au/Pd-TiO_2_ was stronger than that observed for pure TiO_2_. Size and specific surface area (Brunauer-Emmett-Teller surface, BET) of nanoparticles were important factors for toxicity estimation of modified TiO_2_-based nanoparticles. The mechanism of NPs’ cytotoxic action was regarded; the main factor was the release of ions from the TiO_2_ surface, as well as generation of reactive oxygen species and subsequently induced oxidative stress [71].

## 4. Multi-Walled Carbon Nanotubes (MWCNTs)

Certain MWCNTs display asbestos-like toxic effects. To reduce the need for risk assessment, it has been suggested that the physicochemical characteristics or reactivity of nanomaterials could be used to predict their hazard. Fiber-shape and ability to generate reactive oxygen species (ROS) are important indicators of high hazard materials. Asbestos is a known ROS generator, while MWCNTs may either produce or scavenge ROS [73]. Table 3 summarizes the information about nano-(Q)SAR models predicting cytotoxicity of MWCNTs.

Toxicity of MWCNTs—in particular, genotoxicity—was studied in sufficient detail. Quasi-QSAR [76] models were based on the representation of conditions (not on molecular structure) such as concentration, presence of S9 mix (metabolic activation), and usage or not of preincubation in a quasi-SMILES form. Quasi-SMILES descriptor correlation weights were calculated with the Monte Carlo method. The statistical parameters of the models for three random splits—the determination coefficient of the training set R^2^ and the leave-one-out cross validation parameter q^2^—were in the ranges 0.645–0.809 and 0.473–0.726, respectively [74]. In another study with usage of quasi-SMILES descriptors, the model was a function of dose, metabolic activation, and two types of MWCNTs (the first with diameter of 44 nm and surface area of 69 m^2^/g, and the second with diameter of 70 nm and surface area of 23 m^2^/g) [76]. In yet another study, the genotoxicity of MWCNTs was studied, along with fullerenes. Genotoxicity was a function of five parameters: Particle type (fullerene or MWCNT), illumination (dark or irradiation), concentration, metabolic activation, and preincubation. Statistical parameters of the models were satisfactory [78].

Nano-QSAR models were constructed to predict the toxicity of 20 MWCNTs types (276 data records) towards human lung cells by using a quasi-SMILES optimal descriptor [80]. Quasi-SMILES were used to represent the physico-chemical properties and experimental conditions for the MWCNTs: Diameter, length, surface area, in vitro toxicity assay, cell line, exposure time, and dose. The model calculations were performed by using the Monte Carlo method. The quasi-SMILES-based nano-QSAR model provided sufficient statistical parameters (determination coefficient R^2^ for internal validation datasets were in the range 0.60−0.80; R^2^ for external validation datasets were equal to 0.81−0.88).

## 5. Fullerenes

Toropov et al. continued to study the toxicity of fullerenes in further publications. The experimental data on the cytotoxicity of C60 NPs towards *Salmonella typhimurium* was examined [79]. By means of quasi-SMILES descriptors obtained with the Monte Carlo method a mathematical model was constructed. The model was a function of dose, metabolic activation (S9 mix), and illumination (darkness or irradiation). Only one split into the training, calibration, and validation set was made. The statistical parameters of the model were not notably high: R^2^ = 0.755, q^2^ = 0.571 [81]. In the next study, two datasets were used for the bacterial reverse mutation test performed using either *S. typhimurium* or *E. coli* strain WP2 uvrA/pKM101 [79]. By means of the quasi-SMILES optimal descriptors calculated with the Monte Carlo method, mathematical models were built (several splits into the training, calibration, and validation set were made). The models were a function of the same experimental conditions as in the previous study: dose, metabolic activation, and illumination [82]. Table 4 summarizes the information about nano-(Q)SAR models predicting cytotoxicity of fullerenes.

## 6. Silica Nanomaterials

Silica (SiO_2_), or silicon dioxide, is one of the most commonly used ENMs. Silica can be divided into two types: Non-crystalline (amorphous) and crystalline. Amorphous SiO_2_ is also divided into natural amorphous silica and synthetic SiO_2_. SiO_2_ has been studied thoroughly, along with metal oxide NPs, which are discussed above. Here, we concentrate exclusively on silica NPs. Table 5 summarizes the information about nano-(Q)SAR models predicting cytotoxicity of silica nanomaterials.

A predictive model for cytotoxicity of 20- and 50-nm silica nanoparticles was built using so-called quasi-SMILES descriptors as a mathematical function of size, concentration, and exposure time. The calculation was performed using three random splits into training, calibration, and validation sets. Cell viability (%) of cultured human embryonic kidney cells (HEK293) exposed to different concentrations of silica nanoparticles was measured by MTT assay. The models obtained showed that quasi-QSAR and Monte Carlo methods can provide satisfactory models for silica nanomaterials [83].

In one of the studies, numerical data on the cellular viability of silica nanoparticles were taken from literature [85]. These data were randomly split three times into the sub-training, calibration, and validation sets. All models showed high determination coefficients (0.83–0.89), as well as high q^2^ parameter values (0.71–0.82) for the sub-training set. The cv% values were a function of size (20 and 100 nm), concentration, and exposure times (24, 48, and 72 h) [24].

A set of cytotoxicity experimental data corresponding to 19 data points for silica nanomaterials was investigated to compare the widely employed quasi-QSAR (with usage of quasi-SMILES optimal descriptors) and RF approaches. The endpoint of cytoxicity measurement used in this work was EC_25_ (the concentration level which induces 25% of maximum response above the baseline after a given treatment time). The aspect ratio and zeta potential were found to be the two most important variables for RF. Quasi-QSAR failed to reproduce these results. The predictive performance estimated from leave-one-out cross-validation was significantly higher for the RF method and substantially less over-fitting was observed. As a whole, it was demonstrated that the RF approach is applicable to modeling the cytotoxicity of silica nanoparticles [86]. However, later, Manganelli and Benfenati used CORAL software to build nano-QSAR models based on «quasi-SMILES» with more consistent experimental data and high determination coefficients: 0.80–0.95 [87].

## 7. Conclusions

In this study, we reviewed QSAR and SAR studies of several classes of engineered nanomaterials (ENMs): Metal oxides, metal-containing nanoparticles, multi-walled carbon nanotubes, fullerenes, and silica. The relationships between cytotoxicity and ENM properties are complex, tangled, and difficult to understand, which is partly due to the lack of detailed structural data relating to nanomaterials. In this regard, quasi-SMILES appears to be a suitable technique, which allows taking into account the experimental conditions and empirical properties (size, concentration, etc.) of ENMs. On the other hand, experimental artifacts in the characterization of the nanomaterials arise in this case, which is an important factor, in addition to the complicated interactions with living organisms [88].

## Figures and Tables

**Table 1:** Main features of (Q)SAR models predicting cytotoxicity of metal oxide nanoparticles.

Source	Dataset	Endpoint of Cytotoxicity Measurement	*n*	R^2 1^	Software ^2^	Statistical Method	Descriptors
***Escherichia coli***							
[25]	[25]	LD_50_	7	0.979	-	Multiple linear regression (MLR)	Metal cation charge
[20]	[20]	LD_50_	17	0.862	MATLAB	MLR	Enthalpy of formation of a gaseous cation
[26]	[20]	LD_50_	17	0.741–0.838	CORAL	Monte Carlo	SMILES-based optimal descriptor
[27]	[20]	LD_50_	17	0.933	Minitab 16	MLR	Energy gap, hardness, softness, electronegativity, and electrophilicity index
[28]	[20]	LD_50_	17	0.81–0.90	-	MLR	Electronegativity, charge of the metal cation corresponding to a given oxide
[29]	[20]	LD_50_	17	0.93	RandomForest package	Random forest (RF)	S_1_—unbonded two-atomic fragments [Me] … [Me], which were encoded based on Simplex representation of molecular structures (SiRMS)-derived descriptors [34,35], describing distance where potential reaches minimum at van der Waals interactions; rw—Wigner–Seitz radius; ρ—mass density; (CPP)—cation polarizing power; S_2_—SiRMS-derived electronegativity aligned descriptor of oxides molecules—in a sense of the acid-base property of oxides (this parameter increases with a number of oxygens in molecule); S_3_—tri-atomic fragments [Me]-[O]-[Me], which were encoded by SiRMS-derived descriptors, encoding electronegativity; and (SV)—proportion of surface molecules to molecules in volume
[30]	[20]	LD_50_	17	0.955		Ensemble learning	Oxygen percent, molar refractivity, and polar surface area
[31]	[20]	LD_50_	17	-	MATLAB	Read-across	Ionization enthalpy of the detached metal atoms
[18]	[20]	LD_50_	17	0.889–0.982	CORAL	MLR	SMILES-based optimal descriptor
[36]	[20]	LD_50_	16	0.91	-	MLR	Enthalpy of formation of a gaseous cation (ΔH_Me+_), charge of the metal cation (χ_ox_), and pEC_50_ of *HaCaT*
[33]	[20]	LD_50_	16	0.879	SYBYL X1.1 and SPSS statistics v.17	MLR	Enthalpy of formation of a gaseous cation (ΔH_me+_) and polarization force (Z/r)
[37]	[20]	LD_50_	16	0.79	CORAL	Monte Carlo	Quasi-SMILES
[38]	[20]	LD_50_	17	0.92	-	Counter propagation artificial neural network	Metal electronegativity by Pauling scale, number of metal atoms in oxide, number of oxygen atoms in oxide, and charge of metal cation
[39]	[20]	LD_50_	17	0.968	-	RF	Oxygen in weight percentage and enthalpy of formation of a gaseous cation
[40]	[20]	LD_50_	17	0.877 and 0.903	-	MLR and support vector machines (SVM)	HOMO energy, α-LUMO and β-LUMO energy, the average of α-LUMO and β-LUMO, the energy gap between the frontier molecular orbitals ∆E, and molar heat capacity
[8]	[20]	LD_50_	17	0.93	-	Partial least squares (PLS)	Charge of metal ion, metal ion charge-based SiRMS, number of oxygen atoms in brutto formula weighted by ionic potential, covalent index weighted by charge of metal ion, molecular weight of metal oxide weighed by size of nanoparticle, squared thickness of interfacial layer, van der Waals repulsion weighted by size of nanoparticle, and Wigner-Seitz radius weighted by size of nanoparticle
[32]	[32]	LD_50_	17	0.87	Self-written program	MLR	Electronegativity of metal and electronegativity of metal oxide
[41]	[41]	IC_50_	24	-	R	SVM	Conduction band energy and hydration enthalpy (ΔH_hyd_)
**Human keratinocyte cell line (*HaCaT*)**							
[29]	[42]	LD_50_	18	0.96	RandomForest package	RF	S_1_, rw, ρ, (CI)—covalent index of the metal ion, S_2_, and (AP)—aggregation parameter
[31]	[42]	LD_50_	18	-	MATLAB	Read-across	Mulliken’s electronegativity
[42]	[42]	LD_50_	18	0.93	-	MLR	Enthalpy of formation of metal oxide, Mulliken’s electronegativity
[18]	[42]	LD_50_	18	0.961–0.999	CORAL	MLR	SMILES-based optimal descriptor
[36]	[42]	LD_50_	16	0.88	-	MLR	Enthalpy of formation of metal oxide (ΔH_f_) nano-cluster, electronic chemical potential of the cluster, and pEC_50_ of *E. coli*
[37]	[42]	LD_50_	16	0.79	CORAL	Monte Carlo	Quasi-SMILES
[39]	[42]	LD_50_	18	0.918	-	RF	10-based logarithm of solubility measured in mol/L (LogS), topological polar surface area (TPSA), Mulliken’s electronegativity
[8]	[42]	LD_50_	18	0.83	-	PLS	Atom charge-based SiRMS descriptor, charge of the atom weighted by the bond ionicity, charge of metal ion weighted by ionicity of bond, squared ionic potential, ion change-based SiRMS descriptor, number of oxygen atoms in brutto formula per interfacial layer, mass density weighted by ionicity of bond, Wigner-Seitz radius weighted by ionicity of bond, and ionicity of bond based SiRMS
[43]	[42,44,45]	Cell viability (%)	21	-	CORAL	Hierarchical cluster analysis (HCA) and min–max normalization	Quasi-SMILES
**Transformed bronchial epithelial cells (BEAS-2B)**							
[46]	[46]	% of membrane-damaged cells	9	-	Weka	RF	Atomization energy of the metal oxide, period of the nanoparticle metal, nanoparticle primary size, and nanoparticle volume fraction
[6]	[6]	Cell viability (%)	24	-	-	Regression tree	Metal solubility and energy of conduction
[47]	[6]	Cell viability (%)	24	-	RandomForest package	RF	Mass density, covalent index, cation polarizing power, Wigner–Seitz radius, surface area-to-volume ratio, aggregation parameter, and tri-atomic descriptor of atomic charges
[48]	[48]	LD_50_	24	-	RapidMiner	SVM	Conduction band energy and ionic index of metal cation
[49]	[50]	% of membrane-damaged cells	24	0.68	CORAL	Monte Carlo	SMILES-based optimal descriptor, dose, and exposure time
[43]	[6,51,52]	Cell viability (%)	21	0.713–0.733	CORAL	HCA and min-max normalization	Quasi-SMILES
**Murine myeloid cells (RAW 264.7)**							
[6]	[6]	Cell viability (%)	24	-	-	Regression tree	Metal solubility and energy of conduction
[47]	[6]	Cell viability (%)	24	-	RandomForest package	RF	Mass density, molecular weight, aligned electronegativity, covalent index, surface area, surface area-to-volume ratio, two-atomic descriptor of van der Waals interactions, tetra-atomic descriptor of atomic charges, and size in DMEM
[48]	[48]	LD_50_	24	-	RapidMiner	SVM	Conduction band energy and ionic index of metal cation
[53]	[53]	Lactate dehydrogenase (LDH) release	25	-	R	PLS	Metal cation charge, hydration rate, radius of the metallic cation, and Pauling electronegativity
**Rat L2 lung epithelial cells and rat lung alveolar macrophages**							
[54]	[54]	Membrane damage (units L^−1^)	42	-	-	Multivariate linear regression and linear discriminant analysis (LDA)	Size, concentration, size in phosphate buffered saline, size in water, and zeta potential
[55]	[54]	Membrane damage (units L^−1^)	42	-	-	MLR and simple classification	Size, concentration, size in phosphate buffered saline, and size in water

^1^ Missing R^2^ value means that an SAR model was built instead of QSAR. ^2^ If software record is missing, then it was not mentioned in the original paper.

**Table 2:** Main features of (Q)SAR models predicting cytotoxicity of metal-containing nanoparticles.

Source	Dataset	Cell Type	Endpoint of Cytotoxicity Measurement	*n*	R^2^	Software	Statistical Method	Descriptors
[59]	[60]	Monocytes, hepatocytes, endothelial, and smooth muscle cells	Cellular viability	51	0.72	WinSVM, ISIDA	SVM classification and k Nearest Neighbors (kNN) regression	Size, zeta potential, R1 and R2 relaxivities
[59]	[61]	PaCa2 human pancreatic cancer cells, U937 macrophage cell lines, primary human macrophages, HUVEC human umbilical vein endothelial cells	Cellular uptake	109	0.65–0.80	WinSVM, ISIDA	SVM classification and k Nearest Neighbors (kNN) regression	Lipophilicity, number of double bonds
[62]	[60]	Smooth muscle cells	Cell apoptosis	31	0.81	-	MLR and Bayesian regularized artificial neural network	I_Fe2O3_, I_dextran_, and I_surf.chg_
[63]	[60]	Monocytes, hepatocytes, endothelial, and smooth muscle cells	Cellular viability	44	-	-	Naive Bayesian classifier	Primary size, spin-lattice and spin-spin relaxivities, zeta potential
[64]	[64]	Zebrafish embryo	24 h post-fertilization mortality	82	-	ABMiner	Numerical prediction	Concentration, shell composition, surface functional groups, purity, core structure, and surface charge
[65]	[65]	Mammalian cell lines	TC_50_	1681	-	STATISTICA v.6	LDA	Molar volume, polarizability, and size of the particles
[66]	[66]	Algae, bacteria, cell lines, crustaceans, plants, fish, and others	CC_50_, EC_50_, IC_50_, TC_50_, LC_50_	36488	-	STATISTICA	LDA	Molar volume, polarizability, size of NPs, electronegativity, hydrophobicity, and polar surface area of surface coating
[67]	[67]	Bacteria, algae, crustaceans, fish, and others	EC_50_, IC_50_, TC_50_, LC_50_	5520	-	STATISTICA	LDA	Molar volume, electronegativity, polarizability, and nanoparticle size
[68]	[68]	Algae, bacteria, fungi, mammal cell lines, crustaceans, plants, fishes, and others	CC_50_, EC_50_, IC_50_, TC_50_, LC_50_	54371	-	STATISTICA	Artificial neural network	Polar surface area, hydrophobicity, atomic weight, atomic van der Waals radius, electronegativity, and polarizability
[69]	[70]	*Danio rerio*, *Daphnia magna*, *Pseudokirchneriella subcapitata*, and *Staphylococcus aureus*	LC_50_, EC_50_, MIC (minimum inhibitory concentration)	400	-	Weka	Functional tree, C4.5 decision tree, random tree, and CART	Molecular polarizability, accessible surface area, and solubility
[71]	[71]	*E. coli* and Chinese hamster ovary (CHO-K1) cells	EC_50_, MIC	17	0.94	R	Nonlinear least-squaress	Size and specific surface area (Brunauer-Emmett-Teller surface)

**Table 3:** Main features of (Q)SAR models predicting cytotoxicity of multi-walled carbon nanotubes.

Source	Dataset	Cell Type	Endpoint of Cytotoxicity Measurement	*n*	R^2^	Software	Statistical Method	Descriptors
[74]	[75]	*Salmonella typhimurium* TA100	Reverse mutation test TA100	24	0.65–0.81	CORAL	Monte Carlo	Quasi-SMILES
[76]	[77]	*Salmonella typhimurium* TA100	Reverse mutation test TA100	30	0.53–0.64	CORAL	Monte Carlo	Quasi-SMILES
[78]	[77,79]	*Salmonella typhimurium* TA100	Reverse mutation test TA100	44	0.60–0.78	CORAL	Monte Carlo	Quasi-SMILES
[80]	[80]	Four types of normal human lung cells (BEAS-2B, 16HBE14o-, WI-38, and HBE)	Cell viability (%)	276	0.60–0.80	CORAL	Monte Carlo	Quasi-SMILES

**Table 4:** Main features of (Q)SAR models predicting cytotoxicity of fullerenes.

Source	Dataset	Cell Type	Endpoint of Cytotoxicity Measurement	*n*	R^2^	Software	Statistical Method	Descriptors
[78]	[77,79]	*Salmonella typhimurium* TA100	Reverse mutation test TA100	44	0.60–0.78	CORAL	Monte Carlo	Quasi-SMILES
[81]	[79]	*S. typhimurium* TA100	Reverse mutation test TA100	20	0.76	CORAL	Monte Carlo	Quasi-SMILES
[82]	[79]	*S. typhimurium* TA100	Reverse mutation test TA100	20	0.63–0.76	CORAL	Monte Carlo	Quasi-SMILES
[82]	[79]	*E. coli* WP2 uvrA/pKM101	Reverse mutation test WP2 uvrA/pKM101	20	0.68–0.82	CORAL	Monte Carlo	Quasi-SMILES

**Table 5:** Main features of (Q)SAR models predicting cytotoxicity of silica nanomaterials.

Source	Dataset	Cell Type	Endpoint of Cytotoxicity Measurement	*n*	R^2^	Software	Statistical Method	Descriptors
[83]	[84]	Human embryonic kidney cells HEK293	Cell viability (%)	40	0.80–0.93	CORAL	Monte Carlo	Quasi-SMILES
[24]	[85]	Human kidney cells HK-2	Cell viability (%)	42	0.83–0.89	CORAL	Monte Carlo	Quasi-SMILES
[86]	[86]	16HBE, A549, *HaCaT*, NRK-52E, and THP-1	EC_25_	19	0.83	CORAL	Monte Carlo	Quasi-SMILES
[86]	[86]	16HBE, A549, *HaCaT*, NRK-52E, and THP-1	EC_25_	19	0.87	R	RF	Aspect ratio and zeta potential
[87]	[84]	Human embryonic kidney cell line (HEK293)	Cell viability (%)	40	0.80–0.95	CORAL	Monte Carlo	Quasi-SMILES
